# Identification of Material Dimensionality Based on Force Constant Analysis

**DOI:** 10.1021/acs.jpclett.3c01635

**Published:** 2023-08-25

**Authors:** Mohammad Bagheri, Ethan Berger, Hannu-Pekka Komsa

**Affiliations:** Microelectronics Research Unit, Faculty of Information Technology and Electrical Engineering, University of Oulu, Oulu FIN-90014, Finland

## Abstract

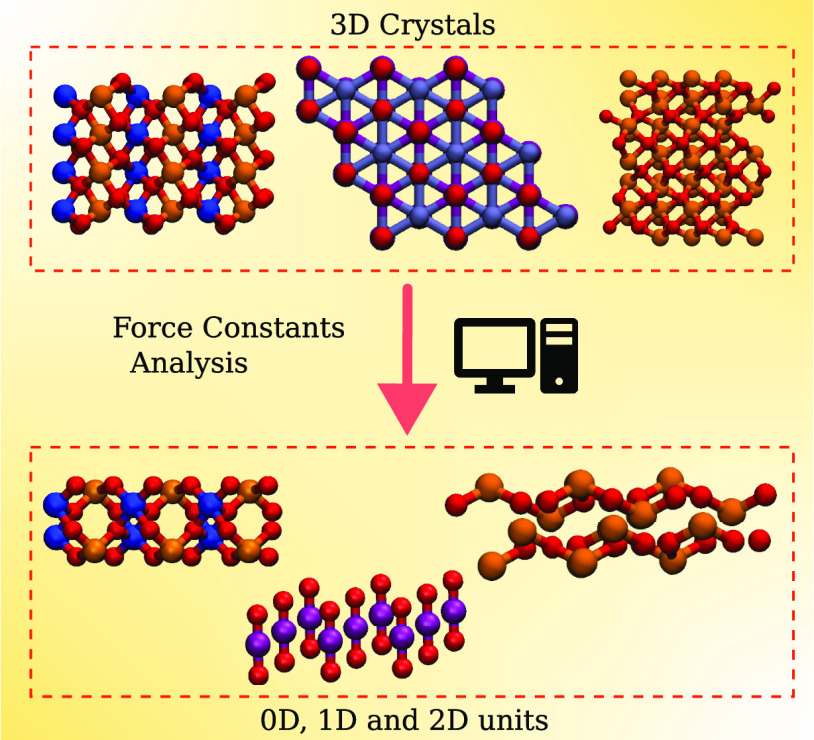

Identification of low-dimensional structural units from the bulk atomic structure is a widely used approach for discovering new low-dimensional materials with new properties and applications. Such analysis is usually based solely on bond-length heuristics, whereas an analysis based on bond strengths would be physically more justified. Here, we study dimensionality classification based on the interatomic force constants of a structure with different approaches for selecting the bonded atoms. The implemented approaches are applied to the existing database of first-principles calculated force constants with a large variety of materials, and the results are analyzed by comparing them to those of several bond-length-based classification methods. Depending on the approach, they can either reproduce results from bond-length-based methods or provide complementary information. As an example of the latter, we managed to identify new non-van der Waals two-dimensional material candidates.

Materials that are easily cleaved in one or more dimensions, such as graphite and other layered materials, have been studied for centuries,^1,2^ but identification and synthesis of the constituent atomic-scale units started with zero-dimensional (0D) Buckminsterfullerene (C_60_)^3^ in the 1980s, one-dimensional (1D) carbon nanotubes (CNT)^4^ in the 1990s, and two-dimensional (2D) graphene in 2004^5^ and have since attracted a great deal of attention. After the potential of these systems was realized, the amount of research dedicated to finding new low- and mixed-dimensional materials with unique electronic,^6−16^ optical,^17−19^ magnetic,^20−22^ and topological^23−26^ properties has remarkably increased. In this context, the “dimensionality” of the material means that the atomic structure consists of units (sheets, clusters, and/or chains) with strong bonds within the unit but weak interactions between the units. Consequently, it becomes possible to extract these low-dimensional units from the parent bulk material with a small energy cost and minor structural changes. There are several computational methods that can identify structure dimensionality and find low-dimensional units in existing bulk materials.^27−32^ These mainly rely on finding the bonds between atoms based on geometrical properties such as atomic positions, atomic radii, coordination of atoms, etc. The great advantage of such methods is that only structural information is required, while the disadvantages are that the methods rely on some heuristic correlation between bond lengths and bond strengths and the atomic radii are averaged from a large set of experimental structures. Nevertheless, they have been demonstrated to be rather successful in identifying 2D materials with small interlayer binding energies.^27,28,30,31^ Naturally, there are also cases in which the correct dimensionality classification is not obvious. For example, quasi-1D materials consist of 1D chains with strong covalent bonds, which are moderately bonded to form 2D layers, which are weakly bonded to form layered three-dimensional (3D) material.^24^ Alternatively, if the interlayer bonds are sufficiently strong, it will be challenging to experimentally exfoliate isolated layers, even if these bonds are clearly weaker than the intralayer bonds. Thus, to make useful predictions, one needs to adopt some practical guidance from the experimental feasibility of exfoliation.

Force constants (FCs) are used to determine the vibrational modes of materials and thus central in many physical properties, such as phonon dispersion, vibrational free energy differences, elastic constants, thermodynamic properties, etc.^33−35^ Importantly, it has been proposed that FCs (albeit with a few slightly different definitions of FCs) provide a good measure of “bond strengths” between atoms.^36−38^ Here, the bond strength is related to the energy change with bond length and is thus a (semi)local property in the potential energy surface (at the equilibrium geometry). This definition avoids problems encountered with bond strengths derived from energy changes upon bond breaking, such as how to treat a changing electronic configuration and whether to use the dissociation energy or barrier.^38^ Because of this useful correspondence, FCs are also used to benchmark bond strength descriptors based, e.g., on the analysis of the total electron density.^39,40^ FCs have also been used to investigate the exfoliability of materials. Khaledialidusti et al.^41^ analyzed the FCs between M–X and M–A bonds in ternary-layered MAX phases and showed the M–A bond is much weaker than M–X bonds, and thus, it should be possible to remove A elements to produce MXenes. Moreover, a correlation between exfoliation energy and the relevant force constants was found.^42^ However, a force constant-based approach for predicting the dimensionality of materials has not been reported.

In this Letter, we study the classification of material dimensionality based on force constants of a large number of systems calculated from first principles. We consider three approaches with different conditions for selecting bonded atoms and compare them to the most common geometry-based approaches. We discuss the origins of the differences and show that some approaches can be used to find new low-dimensional materials, such as the non-van der Waals 2D materials demonstrated herein.

If the potential energy is represented as *U*(*r*_*i*_, ..., *r*_*n*_), where *r*_*i*_ is position of atom *i* and *n* is the number of atoms, then the force on the atom is obtained from the first derivative as , where α is the Cartesian coordinate index and the force constant is the second derivative . In essence, the nondiagonal elements describe the force on atom *j* when atom *i* is moved. The diagonal elements of the force constant matrix describe how the energy changes when only atom *i* moves (while all other atoms remain fixed), and because of the translational sum rule of force constant matrix , it is simply the sum of the nondiagonal force constants in each row or column. To obtain the magnitude of FC between atom pair *ij*, the 3 × 3 tensor is reduced to a scalar value using Frobenius norm . In first-principles calculations, materials are usually treated using periodic boundary conditions with a unit cell or supercell, wherein moving atom *i* means moving also all of its periodic images. If we are interested in force constants arising from moving only a single atom, the supercell must be large enough that the effect from periodic images would vanish. Moreover, following the minimum image convention, only distances up to half the cell size can be employed.

As motivated by refs (36−38) and (40), we assume that a (normal mode) force constant between a pair of atoms serves as a good descriptor for the bond strength. Clearly, the force constant describes only the steepness of the potential energy surface around the equilibrium positions of atoms, but one cannot directly extract the energy change upon bond breaking. Moreover, in many cases, the material would strongly distort upon exfoliation, but this can be described only by explicitly simulating the exfoliation process.

Armed with the force constants between all pairs of atoms, to determine the dimensionality of the structure, we must select a force constant threshold that decides whether a pair of atoms is “bonded”. Before discussing the selection of the appropriate threshold, we first describe our algorithm used to determine dimensionality. The algorithm is illustrated in Figure 1, using 3R-MoS_2_ as an example. The unit cell contains three layers, and the initial supercell size is 4 × 4 × 1. Figure 1a shows forces between selected pairs of atoms with the color denoting the value of the force constant. In the case of MoS_2_, the largest FC belongs to the Mo–S bond with a value of 8.99 eV/Å^2^. Two atoms can be considered bonded when the force constants between them are equal to or larger than the defined threshold *t*. If only these bonds were considered, i.e., selecting *t* slightly below 8.99 eV/Å^2^, this would already lead to the “nominally” bonded MoS_2_ structure shown in Figure 1b. The strongest interlayer FC belongs to the S–S bond at only 0.16 eV/Å^2^, meaning that the layers are indeed weakly bound. Once the bonds are determined, the dimensionality can be determined in largely same way as in previous works.^28,30,31^ Here, an approach similar to that proposed by Ashton et al.^28^ is adopted. We expand the initial supercell and the corresponding force constant matrix to double the size in all directions (Figure 1c). The force constant matrix is expanded using the minimum image convention and setting the long-range FCs that are not included in the initial supercell to zero. We then calculate the connected clusters in the initial and doubled supercell using FC threshold *t* to define connections and generate a graph of connected components with NetworkX.^43^ The clusters are shown in Figure 1 next to the atomic structures, where only the number of connected clusters and the number of atoms in them are meaningful (not their positions in the graph). We note that the FC matrix, and consequently the bonds and the connected graphs, properly account for the material periodicity (e.g., the atoms on the left of the supercell are connected to the atoms on the right), and thus, the number of clusters is independent of the rigid shifts of the atoms within the supercell. The dimensionality of the structure is based on checking the cluster’s periodicity as follows. First, each cluster in the doubled supercell is mapped to the corresponding cluster in the initial supercell. Then, if the number of atoms in the cluster stays the same, it is nonperiodic, but if the number of atoms is ×2, ×4, or ×8, it is periodic in one, two, or three dimensions, respectively. Finally, after the dimensionality is checked for all clusters in the initial supercell, the overall dimensionality of the material can be determined: pure dimensionality of 0D, 1D, 2D, or 3D when all clusters have the same dimensionality or mixed dimensionality, e.g., 01D, 02D, 03D, or 13D, when clusters have different dimensionalities.

**Figure 1:**
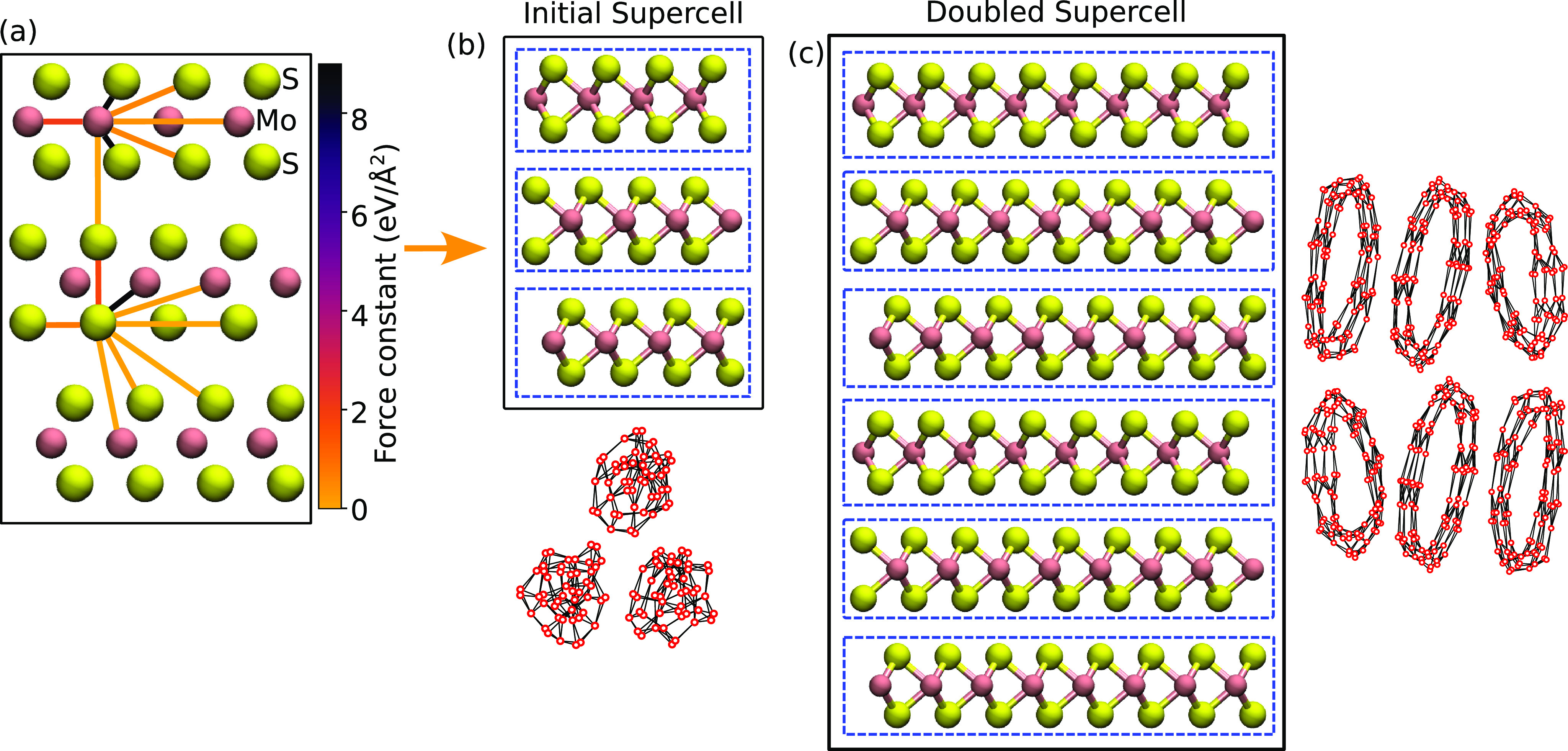
Schematic illustration of the procedure for dimensionality classification. (a) Atomic structure in the initial supercell with selected force constants between pairs of atoms indicated with colored lines. (b and c) Atomic structures and the determined bonds between atoms for the initial and doubled supercell. Dashed line blue boxes show clusters. The graph views of clusters are shown next to the atomic structure, where red circles correspond to vertices (i.e., atoms) and black lines the connections (i.e., bonds).

A choice of a single threshold for all materials could be motivated by, e.g., the observation that the interlayer binding energies of all exfoliatable 2D materials fall close to 13–21 eV/Å^2^^44^ (incidentally having units that match those of the FC). While it is possible to extract such a threshold that correctly classifies simple cases, generally information about the competition between intracluster and intercluster bonding is required, but such information can be extracted from the FC matrix. We first calculate for each atom the maximum (Frobenius-normed) FC:
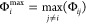
1

From this, we can calculate the maximum and minimum value for each material:
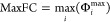
2
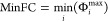
3

MaxFC is the largest (nondiagonal) component of the FC matrix. It will not be useful as a threshold value, because when *t* > MaxFC, there are no bonds between any atoms and all materials are classified as 0D. Selecting the threshold *t* = MinFC (or infinitesimally below it) means that all atoms have at least one bond and is possibly a good choice for threshold.

In addition to selection of a single threshold value *t*, we also consider a scoring scheme motivated by ref (31). We run a scan of *t* from 0 to the MaxFC of the structure to calculate the bonds, clusters, and corresponding dimensionality for each threshold as described above. From the list of dimensionalities as a function of *t*, we then calculate the difference between the maximum threshold (*t*_2_) and minimum threshold (*t*_1_), i.e., *t*_2_ – *t*_1_, for each type of dimensionality. Next, we normalize *t*_2_ – *t*_1_ by the MaxFC of the material to obtain a score within the range [0,1]. In the end, we have a list of dimensionality scores, and the largest score will show the overall dimensionality of the material. As illustrated in Figure 2a for MoS_2_, when 0 < *t* < 0.16 the material is classified as 3D and when 0.16 < *t* < 8.99 the material is classified as 2D. When these ranges are divided by the MaxFC of 8.99 eV/Å^2^, the 2D region affords clearly the highest score of 0.97 and thus the material is classified as 2D.

**Figure 2:**
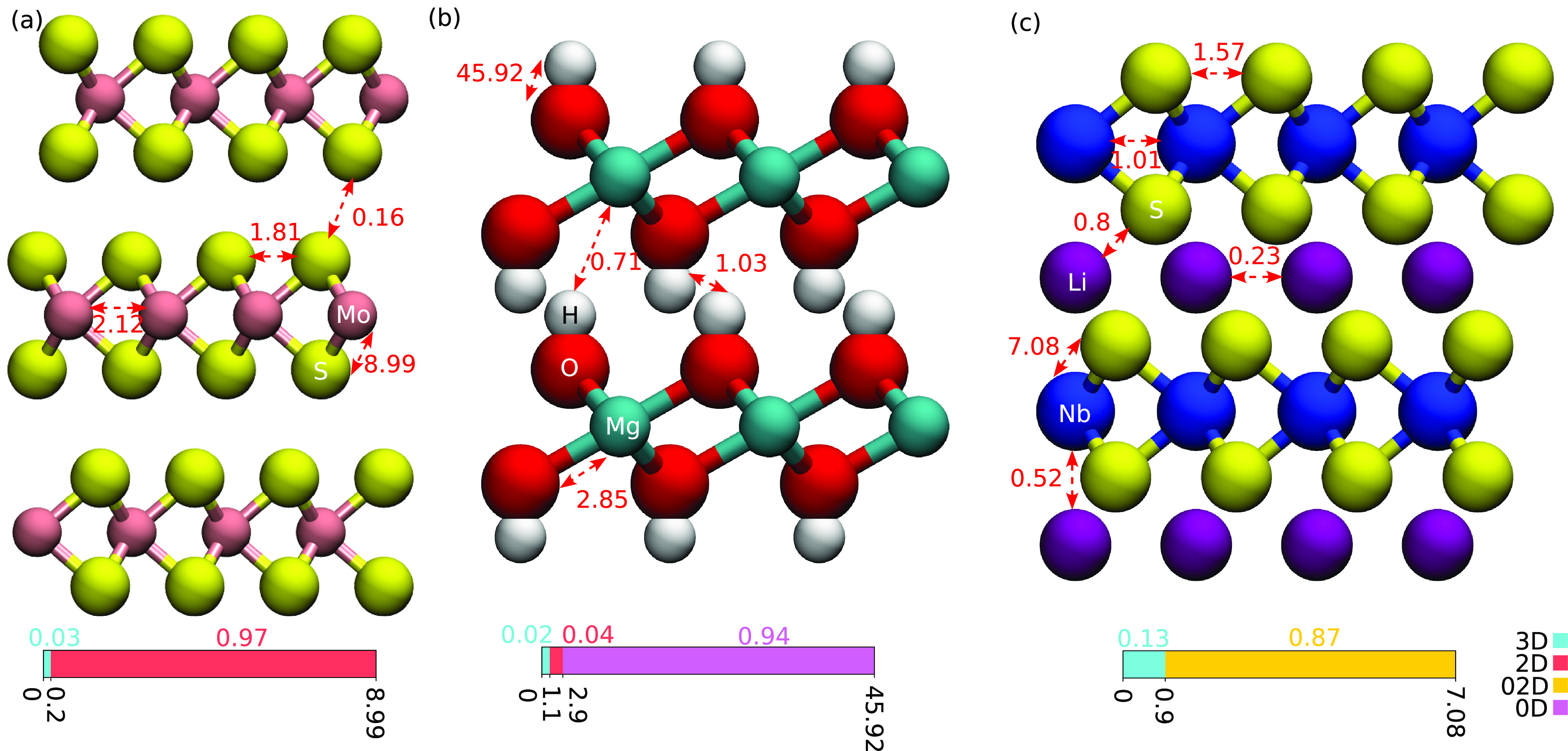
Atomic structures of (a) MoS_2_ (mp-1434) and (b) Mg(OH)_2_ (mp-30247) as two well-known examples of 2D materials and (c) LiNbS_2_ (mp-7936) as a 02D material with Li ions intercalated between NbS_2_ layers. Force constants between selected pairs of atoms are indicated (in eV/Å^2^). Bars under each structure indicate the scores in the scanning approach.

To summarize, we have three approaches for classification: (i) a single fixed threshold, (ii) a threshold based on the force constant matrix, *t* = MinFC, and (iii) a dimensionality score based on the largest *t*_2_ – *t*_1_ range. We illustrate the differences between these approaches using three 2D or 02D materials [MoS_2_, Mg(OH)_2_, and LiNbS_2_], whose structures, selected FCs, and score bars are shown in Figure 2.

As mentioned, MoS_2_ is clearly 2D based on scoring approach iii with a very high score of 0.97. The same is true also with approach ii: Φ_Mo_^max^ = Φ_S_^max^ = MaxFC = MinFC = 8.99 eV/Å^2^. In this case, Mo is bonded to nearest neighbor S, S is bonded to nearest neighbor Mo, and there are no interlayer bonds. Within the fixed threshold of approach i, the material would be classified as 2D for a wide range of *t* values (red bar in Figure 2a). Mg(OH)_2_ in Figure 2b is also expected to be 2D owing to the weak interlayer bonding, and it has been experimentally exfoliated to monolayers.^45^ However, scoring approach iii classifies this material as 0D owing to the much stronger O–H FC compared to the Mg–O FC, and thus for a large range of *t* values, only OH clusters are found (see the score bar in Figure 2b). Within approach ii, however, MinFC is 2.9 eV/Å^2^, which guarantees that all atoms in the Mg(OH)_2_ layers are bonded, and there are no interlayer bonds. In essence, the material would break from “the weakest links”, which is found via MinFC. The choice of a fixed threshold would have to be within the range of 1–3 eV/Å^2^ to obtain 2D classification.

Finally, LiNbS_2_ in Figure 2c would be correctly classified as 02D (2D NbS_2_ layers and 0D Li ions) on the basis of the scoring approach. Because approach ii guarantees that all atoms contain at least one bond, the Li atoms then become connected to the NbS_2_ layers, and consequently, the material is classified as 3D. In the weakest-link view, this is the correct result, because the Li atoms are bonded equally strongly to both layers and thus there is no obvious way to divide the material into clusters with strong intracluster bonds and weak intercluster bonds. In practice, the exfoliation would involve the removal of Li ions and the production of NbS_2_ layers. A fixed threshold of 1–7 eV/Å^2^ would again yield the desired result. This comparison highlights that it will be difficult to select an approach that always produces the desired result, as it will be connected to how the materials will be exfoliated or synthesized.

For a quantitative comparison of different dimensionality classification approaches, they should be applied to a large number of materials, but long-range FCs are rarely included in material databases. Here, we used the data from Atsushi Togo’s Phonon database,^46^ which we also used as a basis for our Computational Raman Database (CRD).^47^ Further calculation details can be found in the Supporting Information. We analyzed all 10 032 materials included in the database and performed the following screening. (i) We checked that the supercell lattice constants are >5 Å. (ii) We checked that the supercell is sufficiently large to contain force constant decay with an increasing atom separation. We required that the force constants for a pair of atoms with a distance along any lattice vector close to half of the lattice constant (due to minimum image convention) be <20% of MaxFC. (iii) We removed three structures for noble gases [Ar (mp-23155), Ar_2_ (mp-568145), and Ne (mp-111)] that have very small force constants, i.e., forces of <0.1 eV/Å^2^. (iv) We checked that the material is dynamically stable; i.e., there are no modes with imaginary frequencies in phonon dispersion. Finally, 4458 materials satisfied these constraints and were used in the dimensionality analysis.

We start with an overview of the data set considered in this work. Figure 3a shows a histogram of the number of structures as a function of MaxFC and MinFC. The maximum force constant value is 110 eV/Å^2^, but the median values of MaxFC and MinFC are 9.63 and 1.71 eV/Å^2^, respectively. Materials with the highest MaxFC have the strongest bonds in the database, and those with the lowest MinFC the weakest. ErCo(CN)_6_ (mp-6185) with a value of 109.4 eV/Å^2^ between C and N atoms and CsAuI_3_ (mp-28453) with a value of 0.12 eV/Å^2^ between Cs atoms have the largest and smallest force constants in the screened materials, respectively. Tables S3 and S4 list 10 materials with the highest MaxFC and lowest MinFC, respectively, and the pair of atoms giving the respective FC. As shown in Table S3, for all 10 materials the maximum FC is found in C–N or N–N bonds. These findings agree with the strongest bonds (N–N bond in [HNNH]^2+^) and materials (C_2_N chains) reported in the literature.^48,49^ The minimum FC (Table S4) is usually found for ions, often alkali metals, that are weakly bonded to the surrounding matrix. Perhaps more interesting are the materials with the lowest MaxFC, which indicates materials in which all of the atoms are weakly bonded, and the highest MinFC, which indicates materials in which all atoms are strongly bonded. These are listed in Tables S5 and S6. The lowest-MaxFC materials are ionic crystals in the rocksalt or antifluorite structure. The highest-MinFC materials are those that are commonly considered hard, such as SiO_2_, but also SiF_4_ molecules, in which all bonds are strong even though the intermolecular bonds are weak.

**Figure 3:**
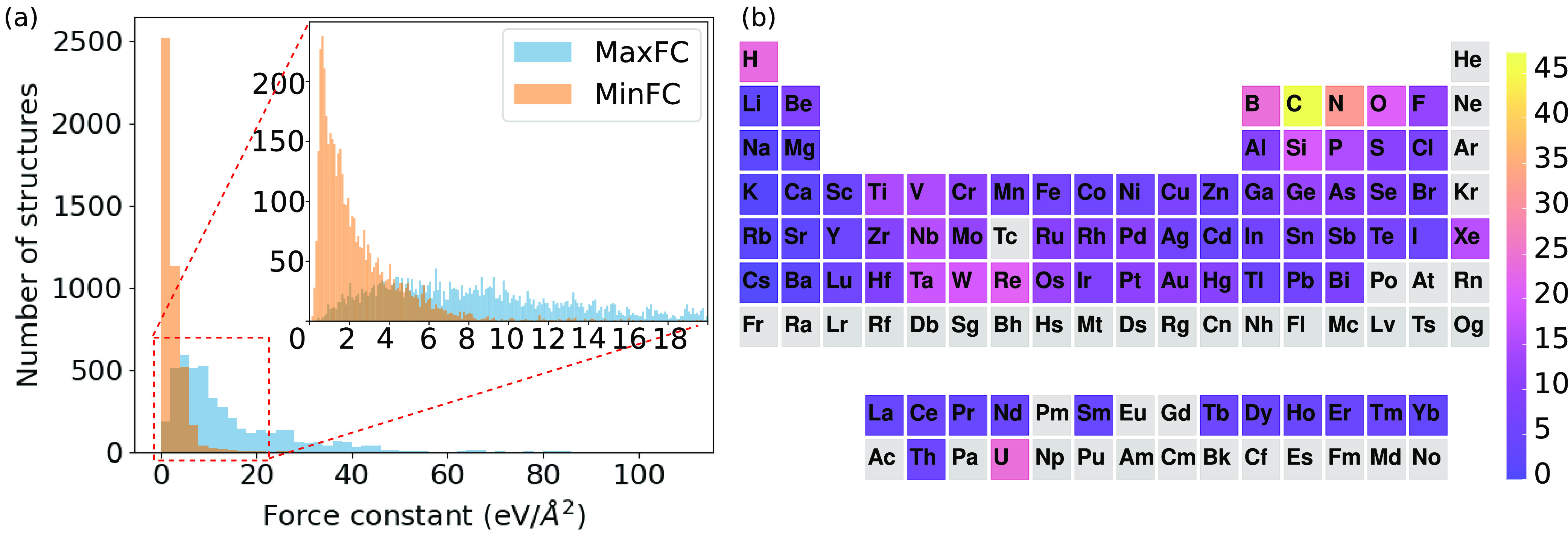
(a) Histogram of the number of screened structures in the data set as a function of MaxFC (blue) and MinFC (orange). (b) Heat map of the average Φ_*i*_^max^ of each element in the data set.

Figure 3b shows the heat map of the average Φ_*i*_^max^ related to each element of the periodic table in the screened materials. The largest values belong to C and N (47.24 and 31.89 eV/Å^2^, respectively). Generally, two “warm” areas can be distinguished: around the organic elements that form strong covalent bonds and the refractory metals (such as Ta, W, and Re) that form very hard materials in combination with C, N, and O. In Figure S3, we show Φ_*i*_^max^ distributions for selected elements. Some of these are clearly unimodal or bimodal, while some can be difficult to classify due to the relatively small number of data points. In a few particularly clear bimodal cases, such as Cu, Si, and Ge, we found the higher peak arose from bonds with oxygen atoms, as highlighted in Figure S3.

The threshold value *t* for approach i is yet to be selected, and for that, we here aim to maximize the match with the Larsen dimensionality. Naturally, one could choose another criterion for selecting *t*, but it is useful to know what *t* would yield agreement with the structure-based classification schemes. Figure 4 shows how the number of structures with a given dimensionality changes with threshold *t*. The best match with Larsen is found around *t* values in the range of 0.4–0.6 eV/Å^2^, depending on the dimensionality. Here, we select a *t* of 0.5 eV/Å^2^, which is our proposed value for fixed threshold classification.

**Figure 4:**
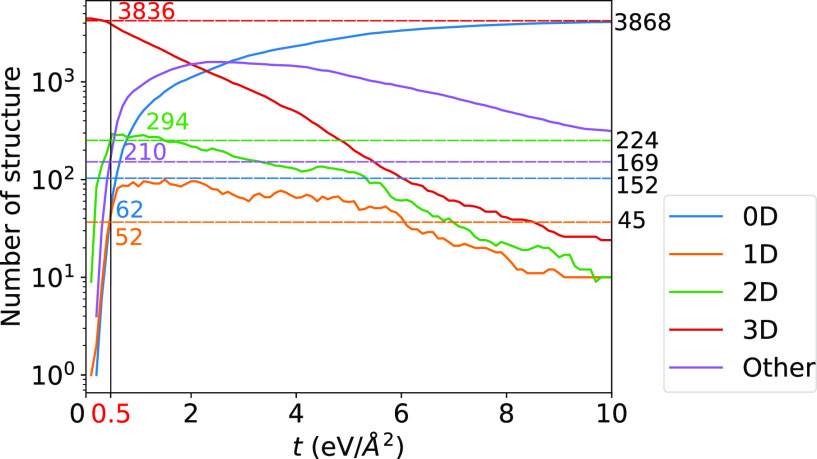
Number of structures grouped by the dimensionality (in log scale) vs threshold *t*. Horizontal dashed lines show the results obtained using the Larsen method. The colored numbers correspond to the number of structures found by our method, and black numbers those found by the Larsen method.

Table 1 shows a comparison of the number of structures with given dimensionality found by our three approaches and the structure-based approaches of Larsen,^31^ Materials Project (robocrystallographer),^50^ Cheon,^29^ and Gorai.^32^ The mixed dimensionality structures are not included in the MP and Gorai methods, and these methods predict only pure dimensionality; also, the Gorai method did not find any 0D structures. Moreover, there were 125 materials for which the Cheon and Gorai methods were unable to determine the dimensionality. Because the Larsen method included any kind of dimensionality (pure and mixed), we used it as the reference method against which our results are compared. Approach i agrees well with Larsen, which may seem obvious but nevertheless shows that good agreement is possible with these different approaches. Approach ii tends to give a larger number of low-dimensional (0D–2D) structures, whereas approach iii gives many more 0D structures arising from a few disproportionately strong bonds within the material (see Figure S1 for plots of *t*_2_ – *t*_1_ vs *t*_1_ for all materials).

**Table 1:** Comparison of the Number of Structures with a Given Dimensionality in the Database as Predicted by Approaches i (with *t* = 0.5 eV/Å^2^), ii, and iii and the Larsen, Materials Project (MP), Cheon, and Gorai Methods^29,31,32,50,51^,^a^

	i	ii	iii	Larsen	MP	Cheon	Gorai
0D	62	580	2581	152	67	51	–
1D	52	564	28	45	75	60	1942
2D	294	910	105	224	295	230	511
3D	3836	2124	692	3868	4021	1234	1880
other	210	280	1052	169	–	2758	–
unknown	–	–	–	–	–	125	125

a Other corresponds to mixed dimensionality structures, and unknown indicates cases in which the method cannot predict the dimensionality.

It is worth noting that a different “normalization” could be applied to approach iii to yield results similar to those of approach i and Larsen. Figure S4 shows the effect of normalization functions  and *ct*/(1 + *ct*) on the number of structures with a given dimensionality based on different values of *c* (similar to Figure 4). With an increase in *c*, the number of 0D structures decreases concurrently with an increase of the number of 3D structures, whereas the numbers of 1D and 2D materials remain largely unchanged. Agreement with Larsen is achieved at around *c* = 1.5 for both functions. Because these results can also be reached using approach i and to keep approach iii free of fitting parameters, we keep the score defined as *t*_2_ – *t*_1_ normalized by MaxFC.

We next take a closer look at materials that were classified differently by our approaches versus that of Larsen. We are here mainly focusing on 2D materials, but we think that this sufficiently demonstrates the typical differences. Figure 5a shows a comparison between the MinFC value used as the threshold in approach ii and the Larsen dimensionality score for all structures classified as 2D by our approach. For the sake of clarity, we removed materials classified as 2D by Larsen’s method but not by our approach ii. The Larsen dimensionality score reflects the range of bond-length thresholds that yield a given dimensionality and is compared to other dimensionality scores to determine the final dimensionality of the material. Clearly, Larsen’s method agrees with our predictions when the dimensionality score is >0.4, but there are many materials with lower scores that are classified as 2D by our approach ii. In particular, we found 304 materials with a Larsen score equal to zero, meaning their method cannot find any 2D unit in these structures.

**Figure 5:**
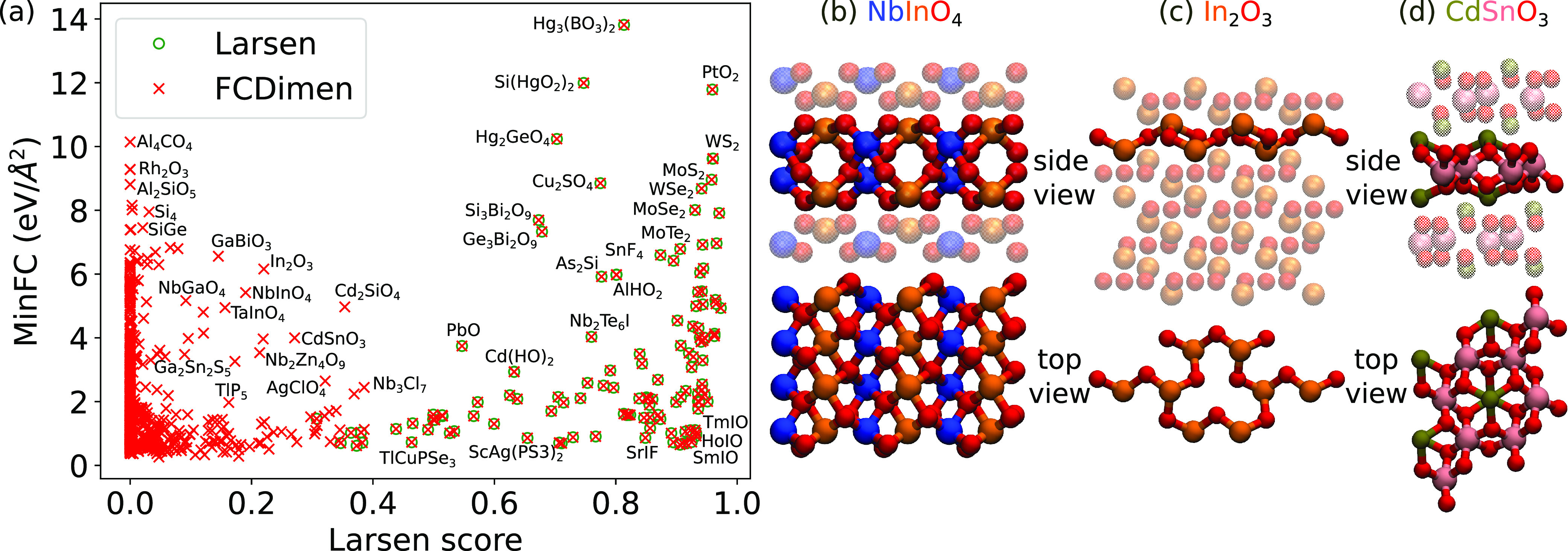
(a) MinFC vs the Larsen dimensionality score for all materials predicted to be 2D using approach ii. Green circles show structures that are predicted to be 2D with the Larsen method, and red crosses structures that are predicted to be 2D with our approach ii. (b–d) Atomic structures (top and side views) of NbInO_4_ (mp-9595), In_2_O_3_ (mp-22323), and CdSnO_3_ (mp-754329), respectively.

To investigate more closely the materials classified differently, we selected three cases that stand out in the plot with a Larsen scores of slightly less than 0.4 and fairly high MinFC values: NbInO_4_, In_2_O_3_, and CdSnO_3_. The atomic structures of the supercell and the extracted 2D materials are illustrated in panels b–d, respectively, of Figure 5, and further information about the labeled materials can be found in Table S1. These materials are not clearly layered with large van der Waals gaps but instead are likely to have fairly strong interlayer interactions. However, on the basis of the FC analysis, the interlayer FCs are still smaller than the intralayer FCs. Indeed, recently Friedrich et al. carried out a data mining study to identify non-van der Waals 2D materials that are structurally similar to the experimentally synthesized hematene Fe_2_O_3_ and ilmenite FeTiO_3_ prototypes.^52,53^ They identified eight binary and 20 ternary oxides, with structures similar to that of CdSnO_3_ in Figure 5d. In the database used in our work, we could find the same four binary materials (Ga_2_O_3_, Al_2_O_3_, In_2_O_3_, and Rh_2_O_3_) and one additional material (Sc_2_O_3_). Curiously, in our case, the 2D material extracted from In_2_O_3_ ended up being only half as thick (Figure 5c) (the ”bilayer” structure is shown in Figure S2). Among the ternary structures, we could find 15 materials, of which 11 are the same and four are new (YBiO_3_, HoBiO_3_, CdSnO_3_, and CaTiO_3_), all having a structure similar to that of CdSnO_3_ shown in Figure 5d. A full list of the mentioned binary and ternary materials can be found in Tables S7 and S8. The important role of structural relaxation in stabilizing this type of monolayers was also discussed in refs (52) and (53), while this aspect cannot be accounted for in our dimensionality classification. Other types of oxides were also identified by our approach, including ABO_4_ ones, such as NbInO_4_ shown in Figure 5b, which, to the best of our knowledge, have not been synthesized in monolayer form. Overall, the predominance of oxides in this set suggests that approach ii is particularly suited for identifying 2D (and other dimensionality) non-van der Waals oxide materials. To verify this, we show in Table S9 the number of oxides found by each approach. Using approach ii, 36% of the predicted 2D structures are oxide materials, compared to 10% and 17% found by approaches i and iii, respectively, and 42% in the whole data set irrespective of dimensionality.

Figure 6a compares dimensionality scores from approach iii and from the Larsen method for materials predicted to be 2D using either of these approaches. A clear separation to three regions can be observed for materials that are identified as 2D by either of the methods or both. We consider in more detail three materials that the Larsen method classifies to not be 2D (YZnASO and LiCuS) and one that approach iii classifies to not be 2D [U(OF)_2_]. The atomic structures are illustrated in Figure 5b–d, and further information about the labeled materials can be found in Table S2. YZnASO and LiCuS are interesting, as the Larsen 2D score is zero or very close to it. YZnAsO actually contains two 2D materials, YO and ZnAs (Figure 6c), and many other similar oxypnictides can be seen in the graph, such as NdZnAsO, LaZnAsO, LaCuSeO, etc. Although the interlayer bonds are relatively short, the corresponding force constants (0.80 eV/Å^2^ for the As–O bond) are markedly lower than for intralayer bonds (4.04 eV/Å^2^ for the Y–O bond and 2.95 eV/Å^2^ for the Zn–As bond) and thus the 2D score is high. In the case of LiCuS, the out-of-plane Li–S FCs (1.20 eV/Å^2^) are much stronger than the interlayer Li–Cu FCs (0.59 eV/Å^2^). Because of the high reactivity of Li, however, it is unlikely that such a 2D material would be stable. A majority of the materials in the bottom right corner of Figure 6a, i.e., identified as 2D by the Larsen method but not by our approach iii, consist of smaller units with very strong force constants, which in turn dominate the dimensionality score. As an example of these, we take U(OF)_2_ (Figure 6b), which is a known layered phase; however, the O–U FCs (54.32 eV/Å^2^) are much stronger than the U–F FCs (2.76 eV/Å^2^), and thus, it is classified as 0D, somewhat similar to the case of Mg(OH)_2_ shown in Figure 2c.

**Figure 6:**
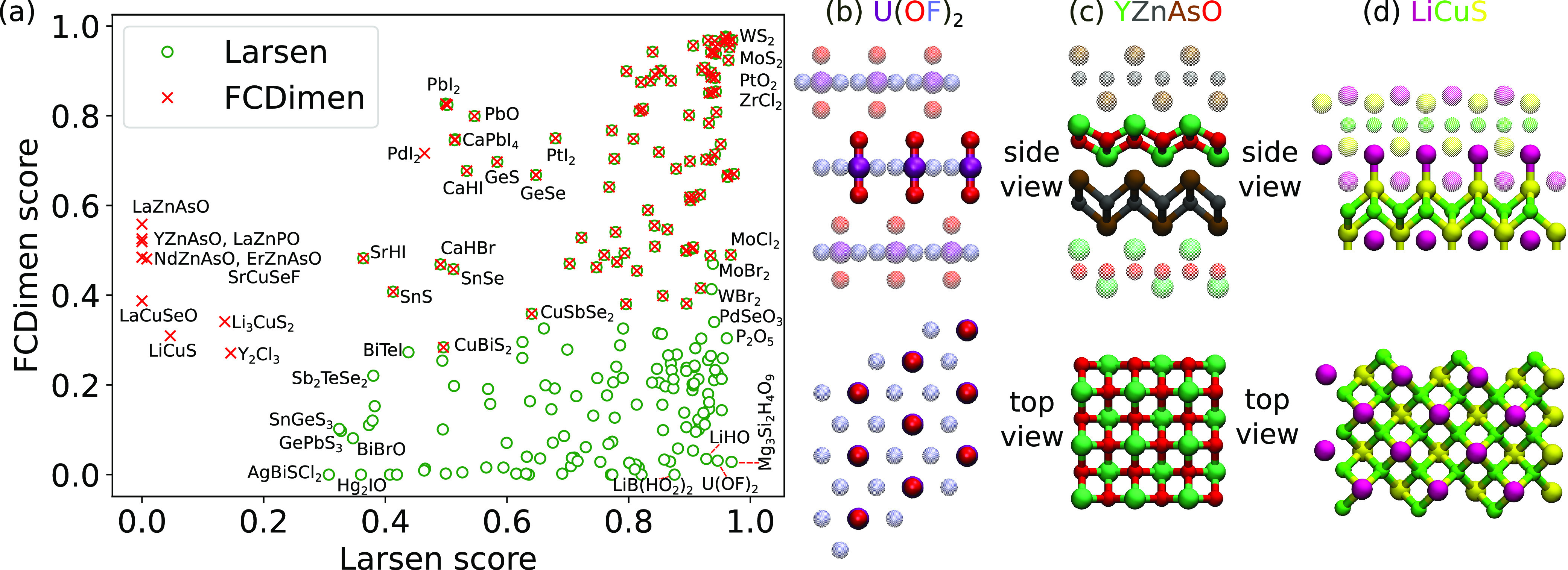
(a) Dimensionality scores from our approach and from that of Larsen for all materials predicted to be 2D using approach iii (red crosses) or the Larsen method (green circles). (b–d) Atomic structures (top and side views) of U(OF)_2_ (mp-27980), YZnAsO (mp-546011), and LiCuS (mp-766467), respectively.

In conclusion, we have introduced a new method for identifying the dimensionality of materials using force constants with three approaches for selecting the bonded atoms. We carried out dimensionality calculations for 4458 materials that include various compound classes, and the calculated dimensionalities are compared to those obtained by existing structure-based methods. For the first approach, we extracted a fixed threshold of 0.5 eV/Å^2^ that can reproduce the results from bond-length methods. The second approach proved to be promising for finding non-van der Waals low-dimensional materials, i.e., those with relatively strong bonding between the units yet even stronger bonding within the units. Finally, the third approach could provide insight into the relative stability of bonds in a material. Importantly, the last two approaches depend only on the calculated force constants and are thus free of any fitting parameters. Thus, each of the three methods can prove to be useful depending on the intended use case.

Calculating force constants is computationally fairly costly, which may limit the applicability of our approach. On the contrary, these calculations could be accelerated by, e.g., using universal machine-learning force fields^54^ or benefiting from the close correlation between FCs and the electron-density-based bond strength descriptors.^39,40^

The approaches demonstrated here are implemented in the open-source package FCDimen.^55^ The whole screened database and the extracted dimensionalities can also be browsed online on the Computational Raman Database Web site (https://ramandb.oulu.fi), where one can also find other relevant information, such as atomic structures, phonon dispersion, and infrared and Raman spectra.

## Supporting Information Available

Figures and tables of the screening procedure, tables comparing the dimensionality prediction of selected structures with those of other methods, and calculation parameters.
