# Supplementary material for: Identification of Material Dimensionality Based on Force Constant Analysis
Source: J Phys Chem Lett. 2023 Aug 25;14(35):7840–7. doi: 10.1021/acs.jpclett.3c01635 (PMC10494234; doi:10.1021/acs.jpclett.3c01635)
Supplement: Supplementary file 1 — jz3c01635_si_001.pdf [file jz3c01635_si_001.pdf]

# Supporting Information for "Identification of Material Dimensionality Based on Force-Constants Analysis"

Mohammad Bagheri, Ethan Berger, and Hannu-Pekka Komsa*

*Microelectronics Research Unit, Faculty of Information Technology and Electrical Engineering, University of Oulu, Oulu, FIN-90014, Finland*

E-mail: hannu-pekka.komsa@oulu.fi

# Methods

The density-functional theory calculations were performed using VASP (Vienna Ab initio Simulation Package)[1,2] with projector-augmented wave method[3] and PBEsol exchange-correlation functional.[4] The plane wave cutoff is set to 1.3 times the maximum cutoff listed in PAW setups. The Brillouin zone of the unit cell is sampled by a $\Gamma$-centered k-point mesh whose density is defined by $R_k = 20$, which in VASP determines the subdivisions $N_1$, $N_2$, and $N_3$ along the reciprocal lattice vectors $b_1$, $b_2$, and $b_3$, respectively, via $N_i = \max(1, R_k|b_i|{+}0.5)$ and rounded to an integer.

Larsen[5] dimensionality calculated with the implemented method in Atomic Simulation Environment (ASE),[6] Materials project (MP)[7,8] dimensionality calculated with robocrystallographer,[9] Cheon,[10] and Gorai[11] dimensionalities calculated with the implemented tool in Pymatgen (Python Materials Genomics).[12]

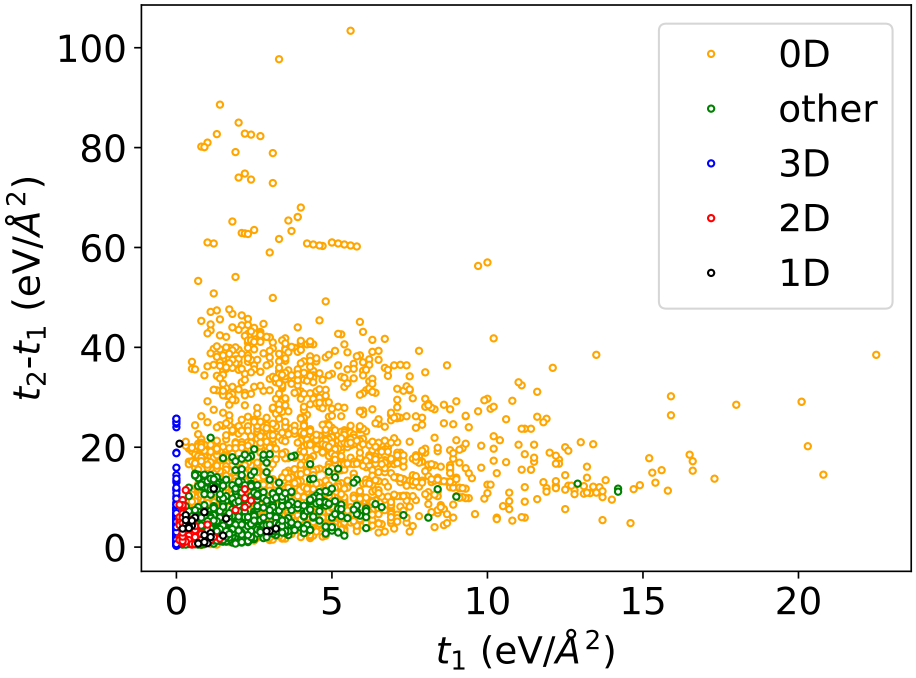


Figure S1: All the analyzed CRD materials indicating FCDimen dimensionality analysis based on threshold scoring system

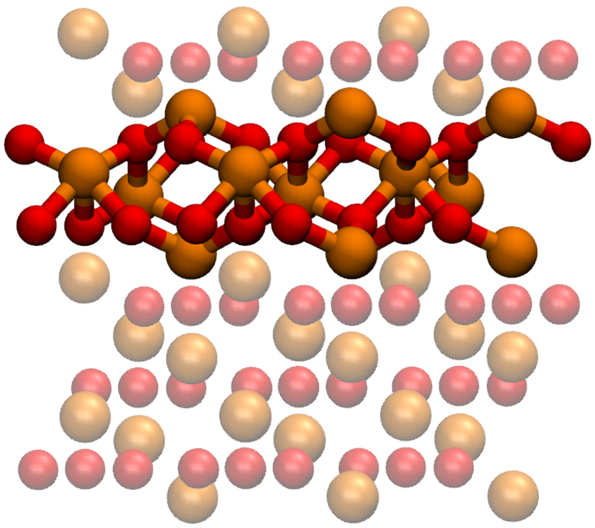

Figure S2: Atomic structure side view of of the bilayer $In_2O_3$.

**Table S1: Comparison of dimensionality prediction of selected structures using classification (ii) with other methods**

| Formula | MPID | FCDimen | Larsen | MP | Cheon | Gorai |
|---|---|---|---|---|---|---|
| $PtO_2$ | mp-7868 | 2D | 2D | 2D | 2D | 2D |
| $WS_2$ | mp-224 | 2D | 2D | 2D | 2D | 2D |
| $WS_2$ | mp-9813 | 2D | 2D | 2D | 2D | 2D |
| $MoS_2$ | mp-1434 | 2D | 2D | 2D | 2D | 2D |
| $WSe_2$ | mp-1821 | 2D | 2D | 2D | 2D | 2D |
| $MoSe_2$ | mp-7581 | 2D | 2D | 2D | 2D | 2D |
| $MoTe_2$ | mp-602 | 2D | 2D | 2D | Unknown | Unknown |
| $SnF_4$ | mp-2706 | 2D | 2D | 2D | 2D | 2D |
| $As_2Si$ | mp-978553 | 2D | 2D | 2D | 2D | 2D |
| $AlHO_2$ | mp-625056 | 2D | 2D | 2D | 2D | 2D |
| $AlHO_2$ | mp-625054 | 2D | 2D | 2D | 2D | 2D |
| $AlHO_2$ | mp-626902 | 2D | 2D | 2D | 2D | 2D |
| TmIO | mp-27439 | 2D | 2D | 2D | 2D | 2D |

**Table S1 – continued from previous page**

| Formula | MPID | FCDimen | Larsen | MP | Cheon | Gorai |
|---|---|---|---|---|---|---|
| HoIO | mp-753173 | 2D | 2D | 2D | 2D | 2D |
| SmIO | mp-754217 | 2D | 2D | 2D | 2D | 2D |
| SrIF | mp-23046 | 2D | 2D | 2D | intercalated ion | 1D |
| $ScAg(PS_3)_2$ | mp-8616 | 2D | 2D | 2D | 2D | 2D |
| $TlCuPSe_3$ | mp-569129 | 2D | 2D | 3D | 2D | 2D |
| $Hg_3(BO_3)_2$ | mp-4710 | 2D | 2D | 2D | 3D | 3D |
| $Si(HgO_2)_2$ | mp-779704 | 2D | 2D | 2D | 3D | 3D |
| $Hg_2GeO_4$ | mp-13995 | 2D | 2D | 2D | 3D | 3D |
| $Cu_2SO_4$ | mp-28491 | 2D | 2D | 2D | 2D | 2D |
| $Si_3Bi_2O_9$ | mp-558672 | 2D | 2D | 3D | 2D | 3D |
| $Ge_3Bi_2O_9$ | mp-29218 | 2D | 2D | 3D | 2D | 3D |
| PbO | mp-19921 | 2D | 2D | 2D | 2D | 2D |
| $Nb_3Cl_7$ | mp-27880 | 2D | 3D | 2D | 2D | 2D |
| $Cd_2SiO_4$ | mp-4530 | 2D | 3D | 3D | 3D | 3D |
| $CdSnO_3$ | mp-754329 | 2D | 3D | 3D | 3D | 3D |
| $In_2O_3$ | mp-22323 | 2D | 3D | 3D | 3D | 3D |
| $NbInO_4$ | mp-9595 | 2D | 3D | 3D | 3D | 3D |
| $Al_4CO_4$ | mp-13703 | 2D | 3D | 3D | 3D | 3D |
| $Rh_2O_3$ | mp-542734 | 2D | 3D | 3D | 3D | 3D |
| $Al_2SiO_3$ | mp-4753 | 2D | 3D | 3D | 3D | 3D |
| LiCuS | mp-766467 | 2D | 3D | 3D | intercalated ion | 1D |

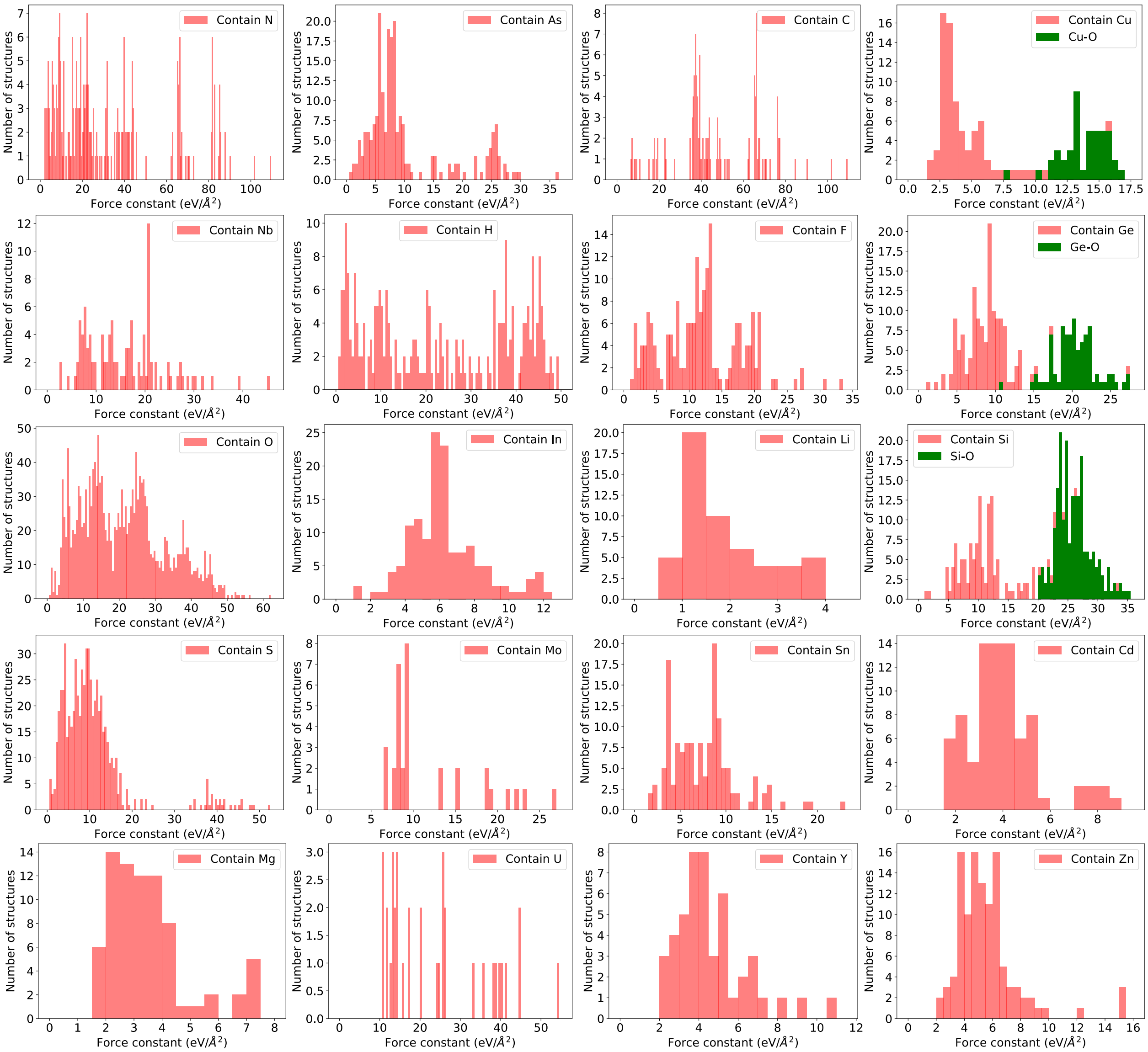


Figure S3: $\Phi_i^{\max}$ distribution of selected elements in the dataset.

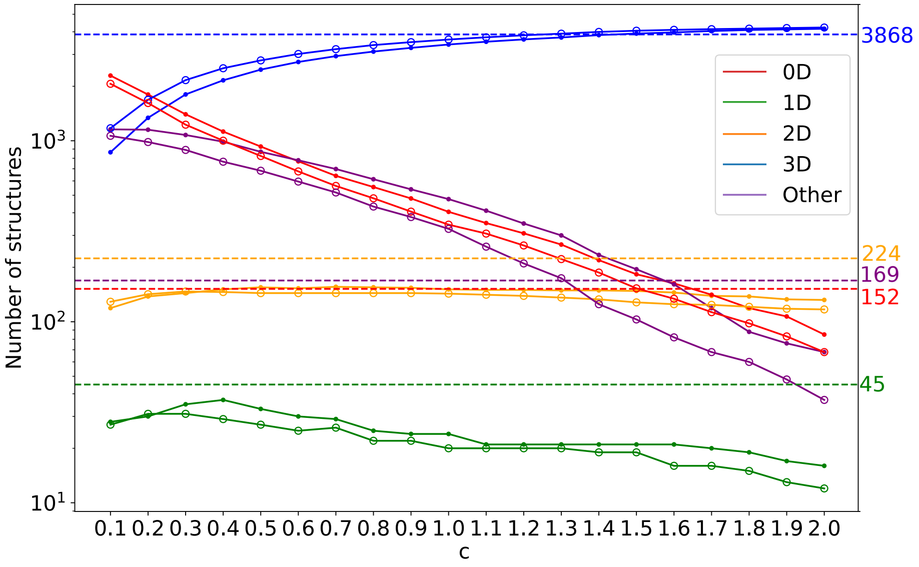


Figure S4: Comparison number of structures predicted using different c parameters in fitting function on approach (iii). solid points are for function $\arctan(ct)/\frac{\pi}{2}$ and empty circles are results using function $\frac{ct}{1+ct}$. Horizontal dashed lines and indicated numbers show the number of structures using the Larsen method.

**Table S2: Comparison of dimensionality prediction of selected structures using classification (iii) with other methods**

| Formula | MPID | FCDimen | Larsen | MP | Cheon | Gorai |
|---|---|---|---|---|---|---|
| $WS_2$ | mp-224 | 2D | 2D | 2D | 2D | 2D |
| $WS_2$ | mp-9813 | 2D | 2D | 2D | 2D | 2D |
| $MoS_2$ | mp-1434 | 2D | 2D | 2D | 2D | 2D |
| $PtO_2$ | mp-7868 | 2D | 2D | 2D | 2D | 2D |
| $ZrCl_2$ | mp-23162 | 2D | 2D | 2D | 2D | 2D |
| $PtI_2$ | mp-28319 | 2D | 2D | 2D | Unknown | Unknown |
| $PbI_2$ | mp-22893 | 2D | 2D | 2D | 2D | 2D |
| $PbI_2$ | mp-567178 | 2D | 2D | 2D | 2D | 1D |
| $PbI_2$ | mp-567503 | 2D | 2D | 2D | 2D | 2D |
| PbO | mp-19921 | 2D | 2D | 2D | 2D | 2D |
| $CaPbI_4$ | mp-754540 | 2D | 2D | 2D | intercalated ion | 1D |

**Table S2 – continued from previous page**

| Formula | MPID | FCDimen | Larsen | MP | Cheon | Gorai |
|---|---|---|---|---|---|---|
| $CaPbI_4$ | mp-753670 | 2D | 2D | 2D | intercalated ion | 1D |
| $PdI_2$ | mp-27747 | 2D | 3D | 2D | 2D | 2D |
| GeS | mp-2242 | 2D | 2D | 2D | 2D | 2D |
| CaHI | mp-24204 | 2D | 2D | 2D | intercalated ion | 1D |
| GeSe | mp-700 | 2D | 2D | 2D | 2D | 2D |
| $MoCl_2$ | mp-620029 | 2D | 2D | 2D | 2D | 3D |
| $MoBr_2$ | mp-29555 | 0D | 2D | 2D | 2D | 3D |
| $WBr_2$ | mp-29498 | 0D | 2D | 2D | 2D | 3D |
| $PdSeO_3$ | mp-545482 | 0D | 2D | 2D | 2D | 2D |
| $P_2O_5$ | mp-2173 | 0D | 2D | 2D | 2D | 3D |
| LiHO | mp-625998 | 0D | 2D | 2D | intercalated ion | 1D |
| $Mg_3Si_2H_4O_9$ | mp-23764 | 0D | 2D | 2D | 2D | 2D |
| $U(OF)_2$ | mp-27980 | 0D | 2D | 2D | 2D | 2D |
| $LiB(HO_2)_2$ | mp-698205 | 0D | 2D | 2D | intercalated ion | 1D |
| SrHI | mp-24205 | 2D | 2D | 2D | intercalated ion | 1D |
| CaHBr | mp-24422 | 2D | 2D | 2D | intercalated ion | 1D |
| SnSe | mp-691 | 2D | 2D | 2D | 2D | 2D |
| SnS | mp-2231 | 2D | 2D | 2D | 2D | 2D |
| $CuSbSe_2$ | mp-20331 | 2D | 2D | 3D | 3D | 3D |
| $CuBiS_2$ | mp-22982 | 2D | 2D | 3D | 3D | 3D |
| LaZnAsO | mp-549589 | 2D | 3D | 3D | 3D | 3D |
| YZnAsO | mp-546011 | 2D | 3D | 3D | 3D | 3D |
| LaZnPO | mp-7060 | 2D | 3D | 3D | 2D | 2D |
| NdZnAsO | mp-7061 | 2D | 3D | 3D | 3D | 3D |
| ErZnAsO | mp-983603 | 2D | 3D | 3D | 3D | 3D |

**Table S2 – continued from previous page**

| Formula | MPID | FCDimen | Larsen | MP | Cheon | Gorai |
|---|---|---|---|---|---|---|
| SrCuSeF | mp-21228 | 2D | 3D | 3D | intercalated ion | 1D |
| LaCuSeO | mp-552488 | 2D | 3D | 3D | 3D | 3D |
| $Li_3CuS_2$ | mp-753737 | 2D | 3D | 3D | intercalated ion | 1D |
| LiCuS | mp-766467 | 2D | 3D | 3D | intercalated ion | 1D |
| $Y_2Cl_3$ | mp-27678 | 2D | 3D | 2D | 2D | 3D |
| BiTeI | mp-22965 | 02D | 2D | 2D | 2D | 2D |
| $Sb_2TeSe_2$ | mp-8612 | 02D | 2D | 2D | 2D | 2D |
| $SnGeS_3$ | mp-5045 | 01D | 2D | 2D | 2D | 2D |
| $GePbS_3$ | mp-624190 | 01D | 2D | 2D | 2D | 3D |
| BiBrO | mp-23072 | 02D | 2D | 2D | intercalated ion | 1D |
| $AgBiSCl_2$ | mp-556094 | 02D | 2D | 3D | 2D | 2D |
| $Hg_2IO$ | mp-28136 | 02D | 2D | 2D | 3D | 3D |

**Table S3: Ten materials with highest MaxFC**

| Formula | MPID | MaxFC | related pairs of atoms |
|---|---|---|---|
| $KN_3$ | mp-827 | 82.56 | N, N |
| $LaCoN_3$ | mp-989643 | 82.85 | N, N |
| $NaHCN_2$ | mp-634434 | 84.81 | N, C |
| $SrN_6$ | mp-2131 | 85.24 | N, N |
| $CaN_6$ | mp-676 | 85.4 | N, N |
| $RbBa_2N_{15}$ | mp-605646 | 85.84 | N, N |
| $BaN_3Cl$ | mp-569812 | 87.77 | N, N |
| NaCNO | mp-546500 | 90.39 | N, C |
| $Cs_2MgFe(CN)_6$ | mp-7331 | 101.93 | N, C |
| $ErCo(CN)_6$ | mp-6185 | 109.37 | N, C |

**Table S4: Ten materials with lowest MinFC**

| Formula | MPID | MinFC | related pairs of atoms |
|---|---|---|---|
| $CsAuI_3$ | mp-28453 | 0.11 | Cs, Cs |
| $K_3PSe_{16}$ | mp-29947 | 0.16 | K, Se |
| CsH | mp-632319 | 0.192 | Cs, H |
| $Rb_3B_{12}H1_2Cl$ | mp-24798 | 0.195 | Rb, Cl |
| $Ge_{19}(PBr)_4$ | mp-27625 | 0.2 | Br, Ge |
| $Rb_3IO$ | mp-29019 | 0.21 | I, O |
| $K_4Au_6S_5$ | mp-29341 | 0.22 | K, S |
| $Ge1_9(AsI)_4$ | mp-27626 | 0.221 | I, Ge |
| $CsAuCl_3$ | mp-23484 | 0.23 | Cs, Au |
| $Rb_7NaGe_8$ | mp-14407 | 0.237 | Rb, Rb |

**Table S5: Ten materials with lowest MaxFC**

| Formula | MPID | MaxFC | related pairs of atoms |
|---|---|---|---|
| CsBr | mp-22906 | 0.34 | Cs, Cs |
| AuCs | mp-2667 | 0.41 | Au, Cs |
| LiI | mp-568273 | 0.43 | I, I |
| CsCl | mp-22865 | 0.45 | Cs, Cs |
| TlCl | mp-23167 | 0.6 | Tl, Tl |
| $K_2Te$ | mp-1747 | 0.64 | K, Te |
| $Rb_2Se$ | mp-11327 | 0.641 | Se, Rb |
| $K_2Se$ | mp-8426 | 0.68 | K, Se |
| CsH | mp-632319 | 0.77 | Cs, Cs |
| CsNaTe | mp-5339 | 0.78 | Te, Na |

**Table S6: Ten materials with highest MinFC**

| Formula | MPID | MinFC | related pairs of atoms |
|---|---|---|---|
| $SiO_2$ | mp-554089 | 25.82 | Si, O |
| $SiO_2$ | mp-7029 | 25.94 | Si, O |
| $SiO_2$ | mp-6945 | 25.95 | Si, O |
| $H_2C$ | mp-985782 | 27.03 | C, H |
| $GeF_4$ | mp-9816 | 27.14 | F, Ge |
| $NbPO_5$ | mp-4888 | 28.77 | O, P |
| $U(HO_2)_2$ | mp-510128 | 31.1 | H, O |
| $SiF_4$ | mp-1818 | 33.41 | F, Si |
| $H_4CN_2O$ | mp-23778 | 38.23 | N, H |
| $OsO_4$ | mp-540783 | 52.73 | Os, O |

**Table S7: List of binary compositions with the same structures as Hematite**

| Formula | MPID | discovered in Ref.[13] |
|---|---|---|
| $Ga_2O_3$ | mp-1243 | * |
| $Sc_2O_3$ | mp-755313 | |
| $Al_2O_3$ | mp-1143 | * |
| $\mathbf{In_2O_3}$ | mp-22323 | * |
| $\mathbf{Rh_2O_3}$ | mp-542734 | * |

**Table S8: List of ternary compositions with the same structures as Ilmenite**

| Formula | MPID | discovered in Ref.[13] |
|---|---|---|
| $TiCdO_3$ | mp-13641 | * |
| $MgGeO_3$ | mp-3759 | * |
| $NaSbO_3$ | mp-4482 | * |
| $NaBiO_3$ | mp-23054 | * |
| $\mathbf{ZnGeO_3}$ | mp-8285 | * |
| $MgTiO_3$ | mp-3771 | * |
| $YBiO_3$ | mp-754152 | |
| $HoBiO_3$ | mp-754300 | |
| $TiZnO_3$ | mp-14142 | * |
| $LiAsO_3$ | mp-9657 | * |
| $\mathbf{CaSnO_3}$ | mp-4190 | * |
| $CdGeO_3$ | mp-8275 | * |
| $\mathbf{CdSnO_3}$ | mp-754329 | |
| $CaTiO_3$ | mp-754701 | |
| $KSbO_3$ | mp-547792 | * |

**Table S9: Comparison of the number of oxides with given dimensionality in the database as predicted by our approaches (1875 oxides of 4458 materials in total dataset (42%)).**

| Dimensionality | (i) | (ii) | (iii) |
|---|---|---|---|
| 0D | 0 of 62 (0%) | 230 of 580 (40%) | 1297 of 2581 (50%) |
| 1D | 2 of 52 (4%) | 217 of 564 (39%) | 3 of 28 (11%) |
| 2D | 30 of 294 (10%) | 326 of 910 (36%) | 18 of 105 (17%) |
| 3D | 1809 of 3836 (47%) | 963 of 2124 (45%) | 174 of 692 (25%) |
| Other | 34 of 210 (16%) | 139 of 280 (50%) | 383 of 1052 (36%) |

# References


(1) Kresse, G.; Furthmüller, J. Efficiency of ab-initio total energy calculations for metals and semiconductors using a plane-wave basis set. *Comput. Mater. Sci.* **1996**, *6*, 15–50.

(2) Kresse, G.; Furthmüller, J. Efficient iterative schemes for ab initio total-energy calculations using a plane-wave basis set. *Phys. Rev. B* **1996**, *54*, 11169–11186.

(3) Blöchl, P. E. Projector augmented-wave method. *Phys. Rev. B* **1994**, *50*, 17953–17979.

(4) Perdew, J. P.; Ruzsinszky, A.; Csonka, G. I.; Vydrov, O. A.; Scuseria, G. E.; Constantin, L. A.; Zhou, X.; Burke, K. Restoring the density-gradient expansion for exchange in solids and surfaces. *Phys. Rev. Lett.* **2008**, *100*, 136406.

(5) Larsen, P. M.; Pandey, M.; Strange, M.; Jacobsen, K. W. Definition of a scoring parameter to identify low-dimensional materials components. *Phys. Rev. Mater.* **2019**, *3*, 034003.

(6) Larsen, A. H.; Mortensen, J. J.; Blomqvist, J.; Castelli, I. E.; Christensen, R.; Dułak, M.; Friis, J.; Groves, M. N.; Hammer, B.; Hargus, C. et al. The atomic simulation environment—a Python library for working with atoms. *J. Condens. Matter Phys.* **2017**, *29*, 273002.

(7) Jain, A.; Ong, S. P.; Hautier, G.; Chen, W.; Richards, W. D.; Dacek, S.; Cholia, S.; Gunter, D.; Skinner, D.; Ceder, G. et al. The materials project: A materials genome approach to accelerating materials innovation. *APL Mater.* **2013**, *1*, 011002.

(8) Ong, S. P.; Cholia, S.; Jain, A.; Brafman, M.; Gunter, D.; Ceder, G.; Persson, K. A. The materials application programming interface (API): a simple, flexible and efficient API for materials data based on representational state transfer (REST) principles. *Comput. Mater. Sci.* **2015**, *97*, 209–215.

(9) Ganose, A. M.; Jain, A. Robocrystallographer: automated crystal structure text descriptions and analysis. *MRS Commun.* **2019**, *9*, 874–881.

(10) Cheon, G.; Duerloo, K.-A. N.; Sendek, A. D.; Porter, C.; Chen, Y.; Reed, E. J. Data mining for new two- and one-dimensional weakly bonded solids and lattice-commensurate heterostructures. *Nano Lett.* **2017**, *17*, 1915–1923.

(11) Gorai, P.; Toberer, E. S.; Stevanović, V. Computational identification of promising thermoelectric materials among known quasi-2D binary compounds. *J. Mater. Chem. A* **2016**, *4*, 11110–11116.

(12) Ong, S. P.; Richards, W. D.; Jain, A.; Hautier, G.; Kocher, M.; Cholia, S.; Gunter, D.; Chevrier, V. L.; Persson, K. A.; Ceder, G. Python materials genomics (pymatgen): a robust, open-source python library for materials analysis. *Comput. Mater. Sci.* **2013**, *68*, 314–319.

(13) Friedrich, R.; Ghorbani-Asl, M.; Curtarolo, S.; Krasheninnikov, A. V. Data-driven quest for two-dimensional non-van der Waals materials. *Nano Lett.* **2022**, *22*, 989–997.
